# The Hippo signaling pathway in pediatric brain tumors: molecular mechanisms and therapeutic opportunities

**DOI:** 10.1007/s10555-026-10349-8

**Published:** 2026-06-13

**Authors:** Maria A. Papanikolaou, Antonios N. Gargalionis, Christos Adamopoulos, Christina Piperi

**Affiliations:** 1 Department of Biological Chemistry, Medical School, National and Kapodistrian University of Athens, 75 M. Asias Street – Bldg. 16, 11527 Athens, Greece; 2 Department of Oncological Sciences, Icahn School of Medicine at Mount Sinai, New York, NY 10029 USA

**Keywords:** Hippo pathway, YAP, TAZ, Pediatric brain tumors, Glioblastoma

## Abstract

Pediatric brain tumors are the second most prevalent malignancy in children with therapeutic options remaining challenging due to vulnerability of the developing brain and the significant side effects of current treatment with targeted therapies offering a promising alternative strategy. The Hippo signaling pathway has emerged as a promising molecular approach since it effectively integrates biochemical and mechanical cues to regulate cell growth and survival, tissue integrity and stem cell behaviour through interaction with major signalling pathways such as Wnt-β-catenin, TGF-β/SMAD, PI3K/Akt/mTOR, GPCR, Notch, Hedgehog and MAPK/ERK. Dysregulation of Hippo signaling has been associated with a broad range of human diseases, including cardiovascular disease, skeletal and neurodegenerative disorders, as well as multiple pediatric malignancies. Activation of the transcriptional co-activators YAP/TAZ–TEAD has proved essential for tumor growth and progression of high-grade pediatric tumors harbouring RTK or histone H3 mutations. Recent data show that YAP activation can distinguish glioblastomas from low-grade tumors in the context of MAPK and PI3K activation while YAP suppression inhibits cell proliferation and tumor growth *in vivo*. Herein, we describe the molecular mechanisms underlying Hippo pathway dysregulation in pediatric gliomas and propose potential therapeutic strategies for effective targeting in different subsets of pediatric brain tumors.

**Graphical Abstract:**

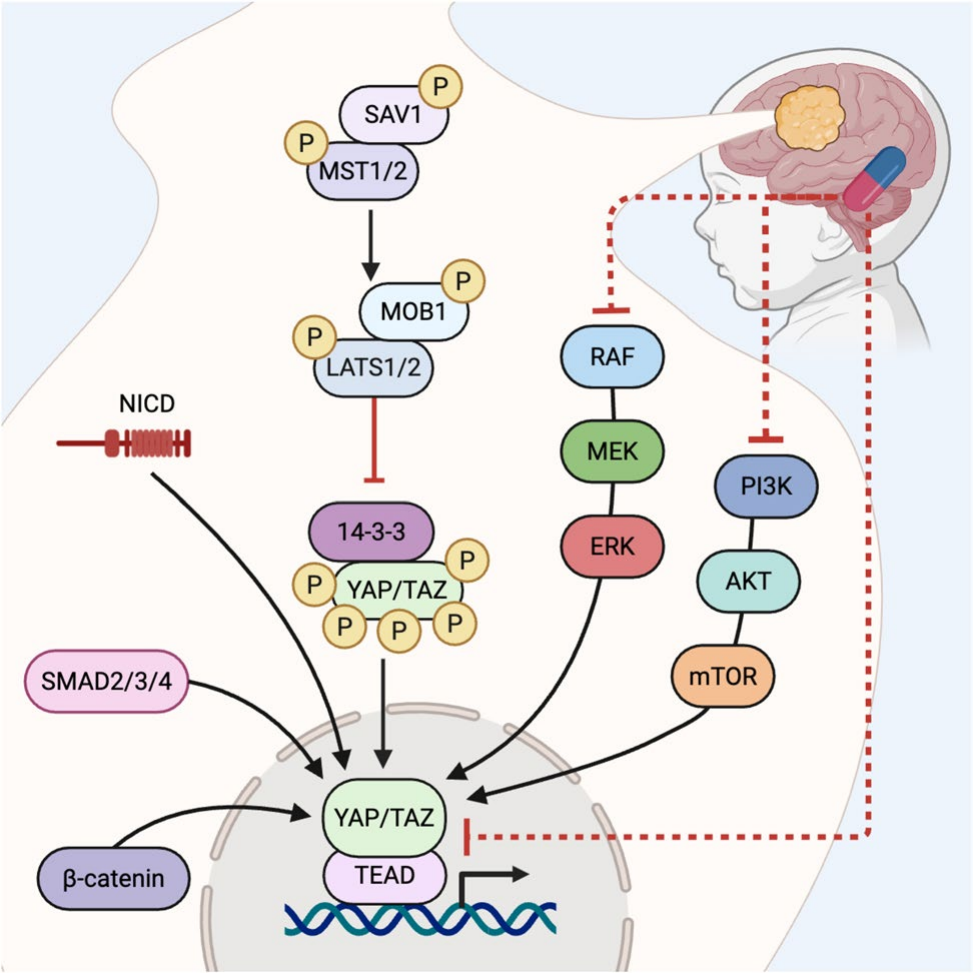

**Supplementary Information:**

The online version contains supplementary material available at 10.1007/s10555-026-10349-8.

## Introduction

Pediatric brain tumors represent the most prevalent solid tumors of childhood and rank second only to leukemia among all pediatric malignancies. Their classification is based on the age of diagnosis with neoplasms identified antenatally or within the first 60 days of life being typically classified as congenital brain tumors, whereas those diagnosed before one year of age being commonly referred to as tumors of infancy [1].

The most frequent brain tumors are gliomas, glioneuronal and neuronal tumors. The 5th edition of World Health Organization (WHO) classification of Central Nervous System (CNS) tumors distinguishes them into six different groups, namely adult-type diffuse gliomas, pediatric-type diffuse low-grade and high-grade gliomas (generally aggressive tumors), circumscribed astrocytic gliomas, glioneuronal and neuronal tumors and ependymal tumors (ependymomas).

Four distinct histomolecular entities are included in the pediatric-type diffuse low-grade gliomas including the angiocentric gliomas, the diffuse astrocytomas with MYB or MYBL1 alteration, the polymorphous low-grade neuroepithelial tumors of the young (PLNTY) and the diffuse low-grade gliomas with altered mitogen-activated protein kinase (MAPK) pathway. On the other hand, the pediatric-type diffuse high-grade gliomas include the high-grade gliomas, H3-wildtype and isocitrate dehydrogenase (IDH)-wildtype, the diffuse hemispheric gliomas with H3G34 mutation; the diffuse hemispheric gliomas with H3K27 mutation and the infant-type hemispheric gliomas, which are frequently characterized by receptor tyrosine kinase (RTK) mutations.

Circumscribed gliomas include pilocytic astrocytomas, pleomorphic xanthoastrocytomas, high-grade astrocytomas with piloid features, chordoid gliomas, astroblastomas, MN1-altered, and subependymal giant cell astrocytomas, MN1-altered. Most circumscribed astrocytic gliomas bear genetic changes in the MAPK pathway.

Ependymomas are distinguished by anatomical site and by specific genetic or epigenetic alterations. They include supratentorial ependymoma, Yes-associated protein-1 (YAP1) fusion–positive; posterior fossa group B (PFB) ependymoma; posterior fossa group A (PFA) ependymoma; and spinal ependymoma, MYCN-amplified [2].

The treatment of brain neoplasms can be challenging and when tumor resection is possible, it is almost always considered as the first line of treatment. Depending on the histological diagnosis and location of the tumor, radiation and/or chemotherapy can be considered as adjuvant therapeutic options while in the case of recurrence, re-resection may also be performed. Combination therapies, such as radiation therapy and chemotherapy can cause significant late effects, due to the vulnerability of the developing pediatric brain. Children younger than 5 years old are especially susceptible to growth arrest, neurocognitive damage, or development of secondary tumors. Molecularly targeted therapies offer the promise of reduced adverse effects in brain tumors; however, they require extensive genetic analysis and investigation of multiple signaling pathways [3–5].

Molecular diagnostics based on next generation gene sequencing have been increasingly applied to provide options for personalized therapies in patients with brain tumors. As previously mentioned, specific signaling pathways are frequently activated in distinct brain tumor types, often due to a range of different genetic alterations, making detailed genetic analysis essential for diagnosing unclear cases. Several tumor groups such as gliomas require a specific molecular analysis as part of the standard pathology workup to enable diagnosis [such as epidermal growth factor receptor (EGFR) amplification as a novel criterion for glioblastoma or 1p19q codeletion to confirm oligodendroglioma] or prognosis and therapy response (such as *MGMT* promoter methylation which determines temozolomide efficacy). For the rarest brain tumors, certain molecular therapies which target key driver mutations can be applied with promising outcome. For instance, the *BRAF* mutations in pleomorphic xanthoastrocytoma (PXA) and papillary craniopharyngioma (PCP), and the tuberous sclerosis complex (TSC) mutations in subependymal giant cell astrocytoma (SEGAs) can be targeted by BRAF inhibitors or mechanistic target of rapamycin (mTOR) inhibitors (such as vemurafenib or everolimus), respectively [6–13].

## Description and normal functions of Hippo signaling pathway

The Hippo pathway is a conserved kinase signaling axis which limits cell proliferation and survival across species, from *Drosophila* (where it was first studied) to humans. Signal transmission is mediated through a tiered serine/threonine kinase cascade in which upstream kinases modulate the phosphorylation state, subcellular localization, and stability of Yes associated protein (YAP)/Transcriptional Co-Activator with PDZ-binding motif (TAZ), thereby controlling transcriptional output [14].

In mammals, the canonical Hippo signaling is initiated upon activation of the Mammalian STE20-like kinases 1/2 (MST1/2) kinases, which undergo autophosphorylation, and dimerization. Subsequently, they phosphorylate Salvador homolog 1 (SAV1), as well as MOB1A/B, thus enabling MOB1 binding to Large Tumor Suppressor 1/2 (LATS1/2) kinases. This interaction facilitates MST1/2-dependent LATS1/2 phosphorylation at conserved hydrophobic motifs, followed by LATS autophosphorylation, resulting in full kinase activation. Activated LATS1/2 phosphorylate YAP and TAZ at multiple conserved serine residues. The phosphorylation at YAP Serine(Ser)127 facilitates attachment to 14–3-3 proteins, causing cytoplasmic retention and functional inactivation [15].

Additional phosphorylation at Ser381 primes YAP/TAZ for sequential phosphorylation by casein kinase 1 (CK1) in a phosphodegron, forming a recognition motif for the SCFβ-TrCP E3 ubiquitin ligase complex. This cascade leads to the ubiquitination and degradation of YAP/TAZ [16]. When Hippo pathway activity is reduced, LATS-mediated phosphorylation of YAP/TAZ is diminished, allowing these proteins to accumulate in an unphosphorylated state and translocate into the nucleus [17].

YAP and TAZ lack intrinsic DNA-binding domains. Therefore, physical interaction and activation of the TEAD family transcription factors 1–4 (TEAD1–4) is critical for the transcriptional function of YAP/TAZ [18]. Some of the best-known YAP target genes include *CTGF, AXL, CYR61,* and *ANKRD1* [19].

TEAD1-4 may act as transcriptional repressors by recruiting Vestigial-like family member (VGLL) proteins, including VGLL3 [20] and VGLL4 [21], which can further compete with YAP/TAZ for binding to TEAD, thereby inhibiting transcriptional activity [22].

The Hippo pathway serves as a fundamental regulator of tissue integrity, organ size, and stem cell behavior by controlling the activity of YAP/TAZ. As a central control point of organ size, Hippo signaling can function to limit cell proliferation and promote apoptosis when required. In *Drosophila*, organ size is controlled through phosphorylation of Yorkie (Yki), the homolog of YAP, at Ser168 with gain-of-function mutations in Yki at this site being lethal at the blastoderm stage. Dong et al. have demonstrated that in mammals, phosphorylation of YAP at Ser127 represents a critical regulatory site involved in growth control. In the liver, YAP upregulation causes reversible hepatomegaly due to excessive hepatocyte proliferation [14].

Similarly, in the developing heart, Hippo signaling limits cardiomyocyte proliferation to control heart size, with deletion of Salvador resulting in increased cell proliferation and cardiac overgrowth [23].

Hippo signaling also has a critical role in the CNS by regulating neural progenitor proliferation. In the chick neural tube, high YAP expression achieved through transduction suppresses differentiation and expands the progenitor cell population, whereas YAP silencing results in elevated apoptosis [24]. In adult hippocampal neural stem cells (NSCs), increased nuclear YAP expression was observed in activated, but not quiescent NSCs. Furthermore, constitutive activation of YAP1 leads to reduced cell-cycle activity and marked expression of mesenchymal cell-type and glioblastoma stem cell-related genes [25].

The Hippo pathway regulates stem cell behavior in multiple tissues, including the intestine, skin, and skeletal system. In intestinal crypts, YAP/TAZ nuclear localization is restricted to crypt base stem cells under physiological conditions, thereby preserving homeostasis. However, during tissue repair, YAP/TAZ can become nuclear in most intestinal epithelial cells and hence, promote proliferation. Furthermore, deletion of YAP/TAZ induces apoptosis, whereas knockdown of *LATS1/2* leads to crypt hyperplasia *in vivo* [26]. In the epidermis, YAP/TAZ are present in the nucleus of basal keratinocytes in both mice and human skin, where they influence gene expression in stem cells. Conversely, in differentiating cells YAP/TAZ is sequestered to the cytoplasm and YAP/TAZ knockdown in mice results in hair loss as well as reduced regenerative capacity [27]. Similarly, Hippo signaling orchestrates chondrocyte proliferation and differentiation during skeletal development. Elevated YAP activity has been shown to promote chondrocyte proliferation and suppress maturation, causing impaired growth and a smaller skeleton in mice [28].

Hippo signaling integrates stimuli such as changes in extracellular matrix (ECM) stiffness to regulate nuclear signaling, thereby allowing cells to adapt to their microenvironment through proliferation and differentiation. ECM stiffening, which causes cell spreading, promotes nuclear YAP/TAZ accumulation and cell growth. Osteoblastic differentiation of mesenchymal stem cells (MSCs), which is normally promoted by a stiff ECM, was shown to be inhibited upon YAP/TAZ knockdown, whereas adipocytic differentiation, which is typically suppressed on stiff ECM, was induced [29]. Furthermore, YAP/TAZ are critical for metabolic homeostasis, with a study of adipocyte LATS1/2 deletion showing increased leptin production and reduced white adipose tissue mass by driving dedifferentiation of mature adipocytes into progenitor-like cells *in vivo* [30]. In skeletal muscle, insulin resistance and decreased fiber size have been associated with YAP downregulation, whereas increased YAP expression was demonstrated to enhance energy consumption and thus, reduce fat content [31, 32].

## Crosstalk between the Hippo pathway and other signaling networks

**Fig. 1:**
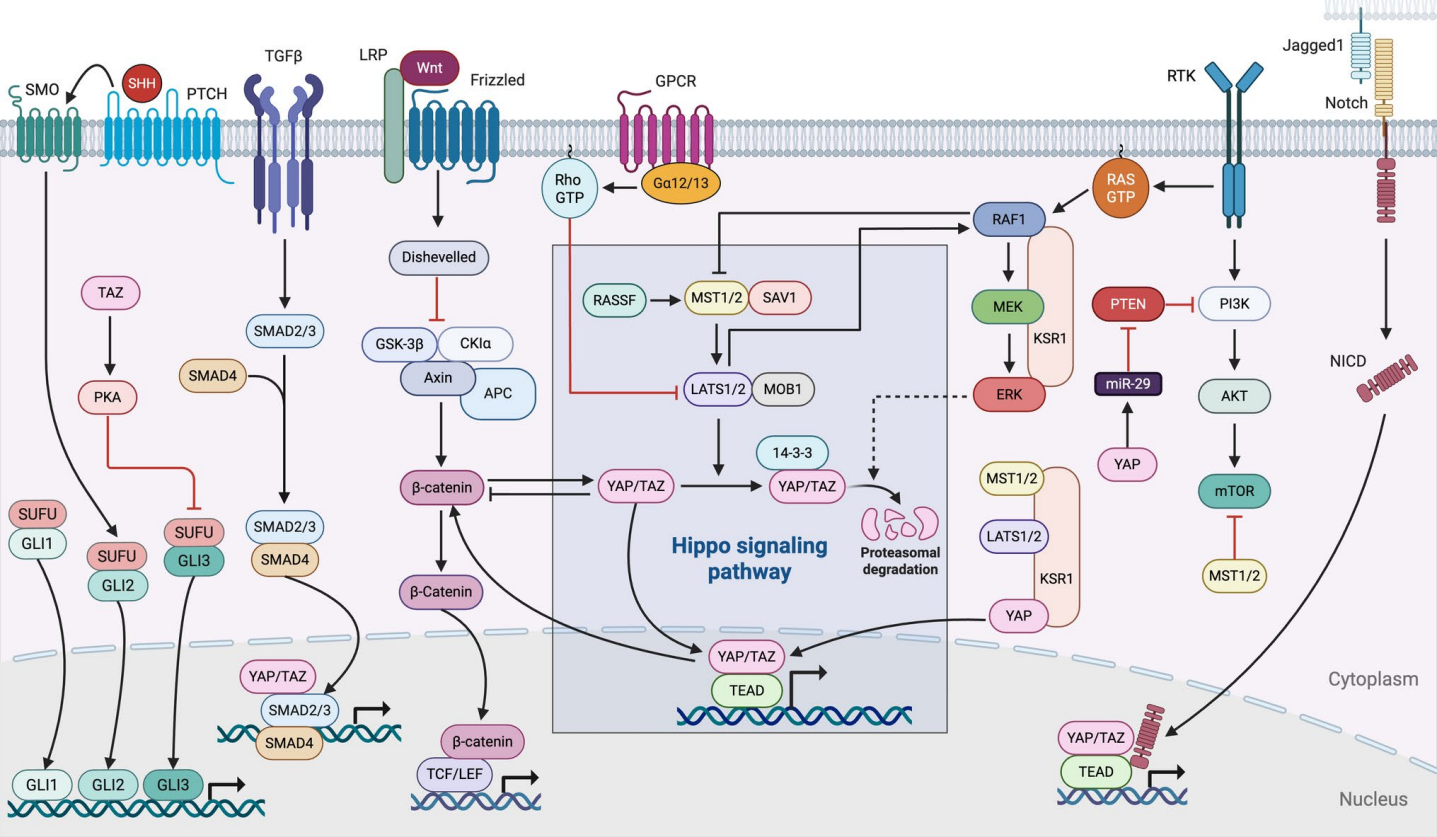
Hippo signaling axis and crosstalk with major oncogenic pathways. Schematic overview of the Hippo pathway core kinase cascade and its crosstalk with developmental and growth-factor signaling. Upstream membrane-associated inputs-including Hedgehog (SHH-PTCH-SMO/GLI), TGF-β (SMAD2/3-SMAD4), Wnt (Frizzled/LRP-Dishevelled-β-catenin), GPCR/Gα12/13-Rho, receptor tyrosine kinases (RTKs; PI3K–AKT–mTOR, RAS-RAF-MEK-ERK), and Notch (Jagged1-NICD)-converge on Hippo components to modulate the activity of the MST1/2-SAV1 and LATS1/2-MOB1 modules. Active LATS1/2 phosphorylates YAP/TAZ, promoting 14–3-3–mediated cytoplasmic sequestration and proteasomal degradation, whereas reduced Hippo kinase activity permits nuclear load of YAP/TAZ and transcriptional activation with TEAD. Crosstalk nodes illustrated include β-catenin-YAP/TAZ interactions, SMAD-YAP/TAZ cooperation, and RTK-driven MAPK and PI3K signaling that can enhance YAP/TAZ output; the PTEN–PI3K axis and miR-29-PTEN regulation are highlighted as modulators of PI3K pathway. The arrows designate activation/forward signaling, blunt-ended lines represent inhibition, and dashed connections indicate indirect or context-dependent regulation; membrane, cytoplasmic, and nuclear compartments are shown. APC, adenomatous polyposis coli; AKT, protein kinase B; CKIα, casein kinase 1α; ERK, extracellular signal-regulated kinase; GLI, glioma-associated oncogene; GPCR, G protein–coupled receptor; LATS, large tumor suppressor kinase; MEK, MAPK/ERK kinase; MOB1, MOB kinase activator 1; MST, mammalian Ste20-like kinase; mTOR, mechanistic target of rapamycin; NICD, Notch intracellular domain; PI3K, phosphoinositide 3-kinase; PKA, protein kinase A; PTCH, patched; PTEN, phosphatase and tensin homolog; RAF/RAF1, RAF kinase; RASSF, Ras association domain family; RTK, receptor tyrosine kinase; SAV1, Salvador homolog 1; SHH, sonic hedgehog; SMAD, suppressor of mothers against decapentaplegic; SMO, smoothened; SUFU, suppressor of fused; TAZ/WWTR1, transcriptional co-activator with PDZ-binding motif; TEAD, TEA domain transcription factor; YAP, Yes-associated protein. Created in BioRender. Adamopoulos, C. (2026) https://BioRender.com/q4jcyq4

There is increasing evidence that Hippo pathway does not act independently, but rather through the interaction with other major signaling pathways (Fig. 1). Crosstalk is highly context-dependent and can be oncogenic or tumor-suppressive depending on tissue type, developmental stage, and microenvironmental cues.

### Wnt/β-catenin crosstalk

Hippo and Wnt pathways exhibit extensive bidirectional regulation. Wnt activation causes an increase in TAZ levels, most probably by enhancing its structural stability, without suppressing the kinases of Hippo pathway (e.g. LATS1/2). YAP/TAZ can physically associate with the β-catenin destruction complex, influencing β-catenin stability. Wnt inactivity causes inhibition of TAZ by β-catenin and its degradation by the β-catenin complex, while Wnt activation and subsequent release of β-catenin from its destruction complex suppresses TAZ degradation. Moreover, transcriptomic analyses showed that over half of Wnt-induced genes are TAZ-dependent. TAZ depletion can inhibit bone differentiation normally promoted by Wnt in stem cells, which suggests that TAZ is heavily involved in Wnt-dependent stem cell differentiation [33]. Wnt also drives YAP nuclear accumulation [34]. Furthermore, overexpression of YAP in intestinal epithelial cells leads to increased β-catenin and Wnt target expression [35], while cytoplasmic YAP/TAZ can repress canonical Wnt signaling [36].

### PI3K–AKT–mTOR signaling

Recent studies have revealed significant crosstalk between Hippo and phosphoinositide 3-kinase/protein kinase B/mTOR (PI3K/AKT/mTOR) pathways. YAP promotes phosphatase and tensin homolog (PTEN) downregulation via miR-29, which blocks PTEN translation, thereby enhancing AKT and mTOR complex 1 (mTORC1) activity [37]. Furthermore, through direct binding, MST1, both in its full and cleaved form, functions as an inhibitor of AKT1. Consistently, reduced MST1/2 levels are associated with increased AKT1 phosphorylation and activity [38]. Conversely, PI3K- phosphoinositide-dependent kinase-1 (PDK1) activation by epidermal growth factor (EGF) or lysophosphatidic acid (LPA) results in the Hippo pathway inhibition and YAP nuclear accumulation *in vivo*, while the application of PI3K or PDK1 inhibitors leads to the cytoplasmic entrapment of YAP [39].

A recent study by Miwa et al. demonstrates the cooperative oncogenic effects between YAP/TAZ and mTOR in various sarcomas, in which dual inhibition of YAP/TAZ–TEAD and mTORC1 produces synergistic antitumor effects [40].

### TGF-β/SMAD pathway

Hippo signaling interfaces bidirectionally with the transforming growth factor-beta (TGF-β)/SMAD, in a highly context-dependent way. During the development of fibrosis, YAP/TAZ play an integral role in the TGF-β1 signaling pathway by interacting with SMADs [41]. In response to TGF-β signaling, TAZ physically associates with SMAD2/3 and 4, promoting their nuclear localization and transcriptional function; in human embryonic stem cells, this results in stem cell renewal, while TAZ knockdown forces neuroectoderm differentiation. However, TAZ phosphorylation and subsequent cytoplasmic sequestration facilitate the cytoplasmic retention of SMADs [42].

Notably, knockdown of *LATS1/2*, resulting in YAP activation, induces TGF-β-dependent epithelial–mesenchymal transition (EMT) in the dorsal neural tube of mice [43].

### GPCR signaling

G-protein coupled receptors (GPCRs) present one of the most potent extracellular regulators of Hippo signaling, by linking several extracellular cues to pathway activity. Ligands like LPA (Lysophosphatidic acid) bind to GPCRs and promote signaling via Gα12/13 to Rho GTPases, which inhibit LATS and thus, facilitate YAP/TAZ activation. Conversely, Gα_s-coupled GPCRs can raise cAMP and activate protein kinase A (PKA), leading to LATS activation and YAP suppression [44].

### Notch signaling

Hippo and Notch pathways interact in mutually reinforcing transcriptional loops. YAP/TAZ interfaces with the Notch intracellular domain (NICD) and thus upregulates Notch ligands such as *Jagged1*. Accordingly, YAP/TAZ knockdown results in diminished Notch pathway activity [45].

Conversely, NICD stabilizes phosphorylated TAZ by preventing its breakdown by β-TrCP.

This positive feedback loop contributes to hepatocarcinogenesis in mice while its disruption can mitigate tumor progression [46].

### Hedgehog (Hh) signaling

The Hippo pathway intersects with Hedgehog signaling through direct regulation of GLI transcription factors. TAZ promotes the PKA-mediated phosphorylation and subsequent repression of GLI3, resulting in inhibition of Hedgehog signaling. In contrast, the cytoplasmic sequestration of TAZ caused by activation of the Hippo pathway induces Hh signaling [47]. Cong and colleagues demonstrated a reciprocal regulatory axis between Sonic Hedgehog (SHH) signaling and YAP during heterotopic bone formation, both of which are upregulated following Gnas loss; SHH is a YAP-driven transcriptional target and SHH secretion in turn induces YAP activation [48]. Similarly, Akladios et al. have shown that Hedgehog signaling promotes YAP upregulation in epidermal skin cells [49].

### MAPK/ERK signaling

MAPK signaling modulates Hippo pathway activity through phosphorylation of upstream kinases (Fig. 1). Raf1, a downstream effector of the MAPK pathway, physically interacts with MST2, thereby inhibiting its activation. Raf1 competes with the tumor suppressor RASSF1A for binding to MST2. Moreover, LATS1 can promote the binding of Raf1 to MST2 through phosphorylation of Raf1 [50].

Knockdown of extracellular signal-regulated kinase 1/2 (ERK1/2) results in increased degradation of the YAP protein and subsequent YAP downregulation, as well as to a decrease in Hippo pathway activity in non-small-cell lung carcinoma cells [51].

In addition, Kinase Suppressor of Ras 1 (KSR1), a Ras scaffold protein which activates the MAPK pathway, has been shown to also act as a scaffold for Hippo, binding directly to YAP, MST1 and forming a complex with LATS1 and YAP. KSR1 can regulate in this manner either the phosphorylation of YAP or the activation of MST1 [52].

## Association of Hippo pathway signaling with disease

Dysregulation of this pathway has been implicated in a range of human diseases, most prominently cancer, but also fibrosis, cardiovascular disease, skeletal disorders, and neurodegeneration. In fibrotic diseases, YAP/TAZ expression is linked to matrix stiffness-dependent profibrotic fibroblast response [53] and myofibroblast activation in organs such as the liver, lung, and kidney, leading to excessive ECM accumulation [54].

In the kidney, podocyte YAP upregulation has been suggested to contribute to glomerular epithelial proliferative disease by inducing a proliferative and profibrotic state in glomerular cells [55].

In the cardiovascular system, YAP1 has been shown to cause cardiomegaly by inducing cell proliferation (i.e., hyperplasia), rather than increasing cell size [56].

Hyperactivation of YAP (via overexpression or LATS1/2 knockdown) in chondrocytes leads to severe chondrodysplasia-like skeletal phenotypes, while YAP/TAZ loss-of-function results in bone structural defects *in vivo*, likely mediated by ECM alterations [57].

Moreover, Hippo pathway imbalance has been associated with neurodegenerative disorders. In amyotrophic lateral sclerosis (ALS), SOD1 mutations can activate MST1, and MST1 inhibition in mouse models reduces symptom severity and slows disease progression [58].

Extensive genetic and functional evidence has established the impact of the Hippo pathway in tumor suppression. Disruption of the pathway contributes to initiation and progression of multiple cancer types through aberrant activation of the YAP and TAZ transcriptional co-activators.

Studies of hepatocellular carcinoma (HCC) have provided some of the most compelling links between Hippo signaling and cancer. While brief activation of YAP in mouse liver induces marked hepatomegaly, prolonged activation led to rapid development of HCC14. Zender and colleagues identified YAP as a key oncogene in 11q22 amplified human HCC and demonstrated that it can function synergistically with the oncogene cIAP1 in driving HCC development [59].

The Hippo pathway dysregulation has also been documented in depth in breast cancer. A study conducted by Cordenonsi et al. discovered a link between high-grade breast carcinomas, metastatic potential and increased YAP/TAZ expression. They also observed that inhibition of TAZ resulted in decreased tumor-forming capacity and that neoplastic breast cells with stem-cell associated traits showed elevated TAZ expression. In addition, TAZ activation could impart stemness to breast cancer cells [60]. With respect to therapy, cohorts studied have shown that high Hippo pathway activity correlates with a diminished response to Trastuzumab and inferior survival outcomes in HER2-Positive breast cancer [61].

In mesothelioma, the upstream regulator of the Hippo pathway NF2 (Merlin), has been established as one of the genes most commonly mutated [62]. Interestingly, Murakami and colleagues also identified LATS2 as a tumor suppressor mutated in a statistically significant percent of mesothelioma cell lines, resulting in proliferation of mesothelioma cells [63].

Aberrant Hippo pathway activity has further been implicated in colorectal cancer, with elevated YAP mRNA and protein levels detected in patient samples relative to normal tissue. YAP upregulation facilitates cancer cell proliferation, migration and possibly EMT *in vitro*, particularly in the context of crosstalk with the AMP-activated protein kinase/cAMP-responsive element-binding protein 1/glucose transporter 3 (AMPK/CREB1/GLUT3) axis [64].

## Impact of Hippo signaling pathway in pediatric tumors

The Hippo signaling pathway has been implicated in the pathology of many pediatric malignancies, with dysregulation of its key-pathway components such as MST1/2, LATS1/2, and the YAP/TAZ effectors having been linked to tumorigenesis and progression of several childhood tumors.

Yang et al. showed immunohistochemically that in patients with neuroblastoma, YAP expression was considerably higher in the neoplastic cells compared to normal tissue in the majority of patients. Additionally, higher YAP expression was associated with a more advanced stage of disease. After using siRNAs targeting YAP on neuroblastoma cell lines, cell proliferation, colony formation and invasion ability were significantly negatively affected. Inhibiting YAP expression in cisplatin-resistant neuroblastoma cells re-sensitized them to cisplatin, which suggests that YAP plays a role in cisplatin resistance [65]. *MYCN*, a gene often amplified in high-risk patients with neuroblastoma, is also positively regulated by TEAD [66, 67].

Aberrant activity of the SHH signaling pathway has been implicated in the formation of medulloblastomas. YAP1 is upregulated in medulloblastoma by the SHH signaling pathway, which is abnormally activated in the SHH-subgroup [68, 69]. YAP1, in turn, stimulates the growth of cerebellar granule neural precursors [70], which are the proposed cells of origin for certain types of medulloblastomas [71].

Cruzeiro et al. showed that YAP1 is overexpressed in medulloblastoma patients with resistance to SMO (Smoothened-Hedgehog pathway) inhibitors compared to patients sensitive to these inhibitors and SHH-like cell lines with *p53* mutations and *YAP1* depletion exhibited improved responsiveness to SMO inhibitors [72].

In embryonal rhabdomyosarcoma, the neoplastic cells are trapped in a constant proliferating myoblast state, even though they express myogenic-differentiation factors [73]. Injecting mice with doxycycline, which induced YAP1 expression in skeletal muscle tissue including myoblasts, led to the rapid development of embryonal rhabdomyosarcoma by suppressing myogenic differentiation [74].

Wilms tumor, also called nephroblastoma, is the most common renal pediatric malignancy and displays loss of the Wilms’ Tumor 1 (*WT1*) gene in a subset of cases [75]. Histologically, tumor cells replicate features of the embryonic kidney. During embryonal development, when YAP/TAZ is phosphorylated and thus held in the cytoplasm, β-catenin signaling activates genes related to nephron induction and differentiation [76]. The cytoplasmic sequestration of phosphorylated YAP is specifically disrupted in anaplastic Wilms’ tumors [77]. He et al. demonstrated via PCR analysis that YAP mRNA is upregulated in Wilms’ tumor tissue compared to normal tissues. Similarly, western blot analysis and immunohistochemistry confirmed that YAP protein expression is upregulated in tumor cells [78].

Ewing sarcoma is an aggressive tumor characterized by the fusion between Ewing sarcoma RNA-binding protein 1 (EWSR1) and most commonly Friend leukemia integration 1 transcription factor (FLI1) and exhibits a low rate of secondary gene alterations [79]. Rodríguez-Núñez et al. showed that YAP1/TAZ are expressed in Ewing Sarcoma cell lines and strong immunohistochemical expression in tumor cells was associated with disease progression. While no known mutations have been found in the Hippo pathway key-components, the Hippo regulator locus RASSF1 is differentially CpG-methylated compared to mesenchymal stem cells. Furthermore, YAP1/TAZ loss-of-function in Ewing sarcoma cell lines reduces cell proliferation and invasion capacity; however, it barely affects migration [80].

## The role of Hippo signaling pathway in pediatric gliomas

Focused studies on the direct impact of the Hippo pathway in pediatric gliomas are limited, partly due to the fact that in earlier decades, many brain tumors were grouped together (e.g. “astrocytoma”, “glioma”) without defined pediatric subtypes, making YAP detection difficult to re-interpret today. Another critical factor to consider is the heterogeneity of pediatric gliomas and the low incidence of certain subtypes, which thereby further complicate the collection of statistically reproducible results from studies.

However, a recent study by Read et al. (2025) used patient-derived tumor cells engrafted into human brain organoid-tumor models to demonstrate that YAP/TAZ–TEAD activity is essential for tumor growth. It was also associated with the progression of high-grade pediatric gliomas with RTK or histone H3 mutations, while the suppression of YAP was shown to inhibit cell proliferation and tumor growth *in vivo* [81]. Additionally, the study of Kim et al. has demonstrated a bidirectional crosstalk between YAP1/TAZ activity and N-cadherin-mediated adhesion in high-grade pediatric gliomas, highlighting that mechanotransduction and cell–cell interactions can cooperatively regulate their invasive behavior [82]. The high-grade-selective dependency of pediatric gliomas on Hippo pathway dysregulation is further supported by an *in vivo* brain tumor model with co-activated MAPK and PI3K signaling, where persistent nuclear YAP activity specifically characterized glioblastomas and distinguished them from low-grade gliomas. Crucially, co-expression of constitutively active YAP with oncogenic RAS exclusively generated high-grade tumors, whereas low-grade glioma development did not require persistent nuclear YAP activity [83]. Although there is a relative lack of studies explicitly comparing Hippo pathway activation between low-grade and high-grade pediatric brain tumors, evidence from pediatric gliomas, medulloblastomas and ependymomas has shown that elevated nuclear localization and transcriptional activity of YAP/TAZ is linked to increased proliferation, stemness, and poorer clinical outcomes [68–72]. In contrast, low-grade tumors generally exhibit more differentiated cellular states, which are typically associated with reduced YAP/TAZ activity. This is consistent with the canonical role of Hippo signaling in restricting growth and promoting differentiation [83–85].

Despite these advances, further pre-clinical studies are demanded to directly investigate the expression and functional role of Hippo pathway in pediatric gliomas. To this end, analysis of single-cell transcriptomics has substantially refined our understanding of pediatric brain tumor biology, particularly by revealing the extent of intratumoral heterogeneity, developmental hierarchies, and microenvironmental interactions that are not captured by bulk profiling.

### Evidence from single-cell analysis studies of pediatric brain tumors

Single-cell RNA-seq studies across pediatric brain tumors consistently show that tumors are composed of hierarchically organized cellular populations that recapitulate aberrant neurodevelopmental trajectories. Specifically, a scRNA-seq analysis of pediatric ependymoma has revealed a continuum from undifferentiated neural stem-like cells to more differentiated neuronal and glial-like lineages, with aggressive tumors enriched in poorly differentiated, progenitor-like populations being associated with worse clinical outcomes [86]. Additionally, integrative single-cell atlases have shown that many pediatric brain tumors arise from lineage-specific progenitors that become “stalled” along normal developmental programs, suggesting that tumor heterogeneity may reflect disrupted differentiation rather than purely genetic divergence [87]. This evidence has a direct implication in Hippo pathway biology, and single-cell analysis in brain tumors has revealed that YAP/TAZ activity is usually enriched in the stem-like or progenitor-like tumor cell populations, where it regulates cellular plasticity and maintains undifferentiated states [88]. Since these undifferentiated states are known to be associated with therapy resistance and tumor recurrences, the Hippo signaling pathway is considered central in sustaining these clinically relevant tumor cell states. Of importance, single-cell approaches may also provide valuable information regarding the tumor microenvironment (TME), and the significant variations in immune and stromal cell populations across pediatric brain tumor types, which are usually different from the respective adult tumors [89]. Since Hippo signaling is responsive to mechanical cues and cellular interactions, it is possible that this spatial and cellular context can modulate YAP/TAZ activity *in vivo*, contribute to functional heterogeneity and influence the clinical translation of Hippo-targeted therapeutic strategies.

Our investigation of the Pediatric Neuro-Oncology Cell Atlas which provides single-cell and single-nucleus RNA-seq data across 19 high-grade and 23 low-grade glioma samples, as well as 26 ependymomas and 28 medulloblastomas, has revealed one study that implicates the Hippo signaling pathway [90]. In this study, Gillen et al. (2020) performed single-cell RNA sequencing of childhood ependymomas and confirmed the presence of a YAP1-Mastermind-like domain-containing 1 (MAMLD1) fusion in supratentorial YAP1-positive (ST-EPN-YAP1) ependymomas. At single-cell resolution, neoplastic cells from this subgroup displayed transcriptional programs enriched for forebrain radial glia and neural stem cell signatures, consistent with the established developmental role of YAP1 in neural progenitor maintenance, and with the clinical observation that ST-EPN-YAP1 tumors arise predominantly in infants and are driven by recurrent YAP1 fusion events [YAP1-MAMLD1, YAP1-Mastermind-like transcriptional coactivator 2 (MAML2), YAP1-family with sequence similarity 118 member B (FAM118B)].

These single-cell data further substantiate the molecular specificity of YAP1 dysregulation in pediatric CNS tumors and its relevance to the neural developmental context. In addition, the Childhood Cancer Data Lab’s Single-cell Pediatric Cancer Atlas (ScPCA) can also provide curated, processed single-cell and single-nucleus RNA-seq datasets from a broad range of pediatric malignancies and needs to be exploited in future studies.

### Possible mechanisms linking the Hippo pathway to pediatric gliomas

Although there are currently limited data in pediatric gliomas, several mechanisms that dysregulate the Hippo pathway in adults are likely to be applied in pediatric gliomas as well.

**Fig. 2:**
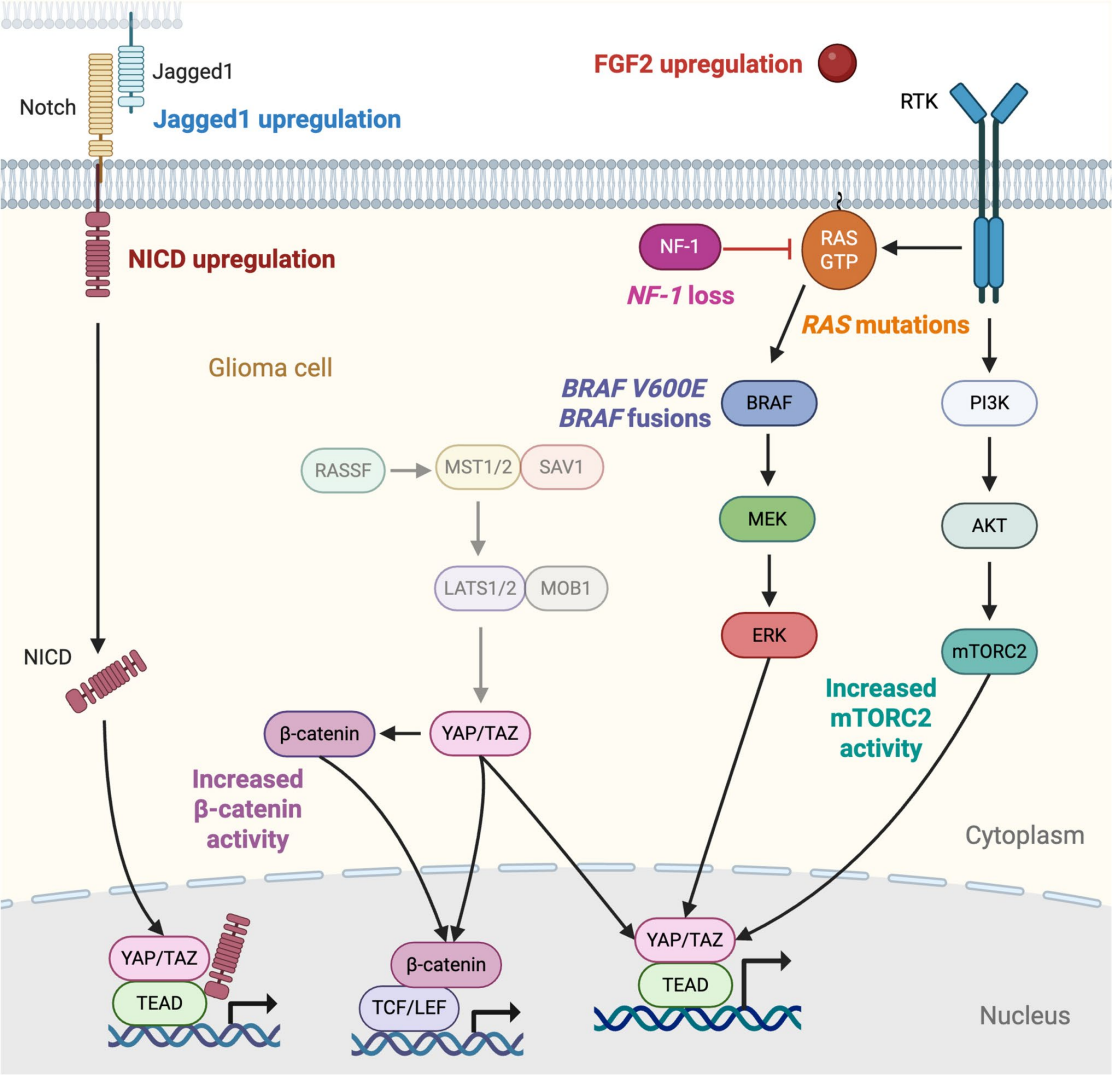
Possible mechanisms linking Hippo-YAP/TAZ signaling to pediatric gliomas. Conceptual model summarizing candidate genetic and microenvironmental drivers that may elevate YAP/TAZ transcriptional output in pediatric glioma cells. Upstream oncogenic signaling is depicted through RTK activation and RAS pathway induction, including NF1 loss (relieving negative regulation of RAS), *RAS*-activating mutations, *BRAF V600E* mutation and *BRAF* fusions feeding into the MAPK cascade (BRAF–MEK–ERK). In parallel, RTK-dependent PI3K signaling activates AKT converging on mTORC2, which may further promote YAP/TAZ activity. Developmental pathway inputs are also highlighted, including Jagged1/Notch activation leading to NICD upregulation, with downstream reinforcement of YAP/TAZ–TEAD transcriptional programs. Increased β-catenin activity (Wnt pathway output) is shown cooperating with YAP/TAZ and promoting β-catenin/TCF–LEF transcriptional responses, providing a second route to pro-growth gene expression. The canonical Hippo kinase module (RASSF–MST1/2–SAV1–LATS1/2–MOB1) is indicated as an inhibitory brake on YAP/TAZ; attenuation of this brake or strong upstream mitogenic signaling is proposed to favor nuclear YAP/TAZ–TEAD activity. Arrows indicate activation/flow of signaling; inhibitory links and convergence on YAP/TAZ emphasize potential points of therapeutic interception. BRAF, B-Raf proto-oncogene; AKT, protein kinase B; ERK, extracellular signal-regulated kinase; FGF2, fibroblast growth factor 2; LATS, large tumor suppressor kinase; MEK, MAPK/ERK kinase; MST, mammalian Ste20-like kinase; MOB1, MOB kinase activator 1; mTORC2, mechanistic target of rapamycin complex 2; NICD, Notch intracellular domain; NF1, neurofibromin 1; PI3K, phosphoinositide 3-kinase; RAS, RAS GTPase; RASSF, Ras association domain family; RTK, receptor tyrosine kinase; SAV1, Salvador homolog 1; TEAD, TEA domain transcription factor; TCF/LEF, T cell factor/lymphoid enhancer factor; YAP, Yes-associated protein; TAZ/WWTR1, transcriptional co-activator with PDZ-binding motif. Created in BioRender. Adamopoulos, C. (2026) https://BioRender.com/wyd5lv0

First, there is evidence of YAP/TAZ activation downstream of common oncogenic drivers in pediatric gliomas (Table 1). As previously described, many pediatric gliomas harbor mutations/fusions that activate MAPK (e.g. *BRAF* fusions, *BRAF V600E* mutation, *NF1 *loss) or PI3K–AKT/mTOR signaling. Mayrhofer et al. demonstrated in a zebrafish model of brain tumors with MAPK and PI3K activation that persistent nuclear YAP activity can distinguish glioblastomas from low grade gliomas. Of note, co-expression of constitutively active YAP with oncogenic RAS exclusively generated high-grade tumors, whereas the development of low-grade glioma did not require persistent nuclear YAP activity (Fig. 2) [91].

Furthermore, Holmes et al. proved that in glioblastoma cell lines mTORC2 activity enhances YAP transcriptional activity, thus increasing tumor growth and invasion [92].

Studies have shown that human glioma cells express higher levels of YAP and knockdown of YAP reduces *in vitro* cell proliferation and intracranial glioma growth in mice. This is partly because of YAP effect on β-catenin. Wang et al. demonstrated that overexpression of YAP leads to an increase of β-catenin activity and its nuclear localization while down-regulation of YAP achieves the opposite effect [93].

YAP also interacts with the Notch signaling pathway, previously shown to promote glioma cell survival, proliferation and invasion. It has been demonstrated that overexpression of YAP1 in glioma cell lines leads to increased metastatic potential via activation of the Notch pathway [94]. More specifically, YAP1 overexpression correlates with upregulation of the Notch ligand Jagged1, the intracellular domain of the Notch protein and Hairy and Enhancer of Split 1 (HES1) (a downstream target gene of the Notch pathway that prevents cell differentiation) [95–98].

There is evidence that YAP/TAZ-TEAD signaling is also involved in neural stem/progenitor proliferation and suppression of differentiation. While the role of glioma stem cells (GSCs) has been thoroughly investigated in adult gliomas, where it has been linked to increased proliferation and invasion [99, 100], less information is available in the literature about GSCs in pediatric brain tumors. The importance of GSCs in these tumors has been proven for the most part in diffuse midline gliomas with H3K27 mutation, in which most neoplastic cells are frozen in the stage of oligodendrocyte precursor cells [101]. A promising study by Hemmati et al. confirmed the existence of tumor-derived progenitor cells in a limited group of pediatric patients with brain tumors including gliomas [102].

Several studies further demonstrate a potential contribution of Hippo pathway to therapy resistance, survival, and recurrence. As previously mentioned, the first line of treatment for gliomas, especially diffuse and high-grade ones, includes radiotherapy and chemotherapy after surgical excision of the tumor. Zhang et al. studied the implication of the Hippo pathway in the radioresistance of gliomas and discovered a link between high YAP expression in patients undergoing radiotherapy and adverse prognosis in databases. However, glioma cells after exposure to radiation did not express high levels of YAP; nonetheless, the protein levels of AXL and Cyr61, two target genes of YAP, were affected. Notably, YAP nuclear levels increased after radiation; this finding suggests radiotherapy induced nuclear localization of YAP. Glioblastoma cells overexpressing YAP exhibited increased colony formation after radiation exposure and decreased radiosensitivity *in vitro*. In addition, YAP overexpressing cells showed increased levels of DNA repairing proteins and less DNA damage after radiation. The underlying mechanism responsible for the development of radioresistance was suggested to be the upregulation of fibroblast growth factor 2 (FGF2) which is involved in the activation of the MAPK-ERK pathway. Moreover, the inhibition of YAP-FGF2-MAPK resulted in reduced tumor growth and increased survival [103].

The Hippo pathway can also influence therapeutic response to temozolomide. Tian et al. demonstrated that resistant to temozolomide cell lines expressed higher levels of TAZ and that sensitive to temozolomide cell lines became more resistant after overexpression of TAZ. In fact, a reduction in apoptotic activity was observed in cells overexpressing TAZ, which was attributed to upregulation of Myeloid cell leukemia 1 (MCL-1), an apoptosis-suppressing BCL-2 family protein. Moreover, knockdown of *MCL-1* considerably mitigated the anti-apoptotic effect of YAZ on cells [104].

Although TEAD transcription factors are the principal DNA-binding partners of YAP and TAZ, the oncogenic effects of YAP/TAZ–TEAD signaling are largely affected by cooperation with other lineage- and context-specific transcriptional regulators. Genome-wide studies have shown that YAP/TAZ–TEAD complexes can co-occupy enhancers with AP-1 family members, activating gene transcription responsible for cell-cycle progression, proliferation, and motility [105, 106]. This is the case for MAPK-driven pediatric gliomas, in which AP-1 activity is increased and is converged with YAP/TAZ-dependent transcription [105, 106]. Additionally, in breast cancer cells, YAP/TAZ has been shown to interact with SMAD2/3 downstream of TGF-β signaling, maintaining a tumorigenic and metastatic phenotype [105, 106]. The Wnt/β-catenin signaling pathway can also shape YAP/TAZ output through β-catenin/TCF–LEF-dependent transcription, a mechanism that could be relevant in brain tumor development [34]. In medulloblastoma, YAP1 has been linked to Sonic Hedgehog-driven neural precursor proliferation, suggesting functional interaction with SHH/GLI-dependent programs [70]. Moreover, a tissue-specific cooperativity of TEAD has been shown in MYCN-associated neuroblastoma [67]. Table 1.

**Table 1:** Proposed Hippo signaling involvement in different types of pediatric gliomas

Tumor Class	Tumor examples	Key molecular drivers	Involved molecular pathway	Proposed Hippo pathway involvement	Crosstalk with other pathways	Reference
Circumscribed gliomas	Pilocytic astrocytoma	BRAF fusion (KIAA1549–BRAF), *BRAF V600E* mutation, *FGFR1, NF1* mutations	MAPK/ERK	**Moderate (Pathway crosstalk)**-BRAFV600E and KIAA1549-BRAF fusion driven MAPK activation can affect LATS 1/2 phosphorylation status -CRAF/RAF1 inhibits MST2 activation	-PI3K–AKT–mTOR pathway activation, especially in clinically aggressive or anaplastic cases of pilocytic astrocytoma, may enhance YAP/TAZ activity	[107–109, 117]
Pediatric-type diffuse low-grade gliomas (pDLGGs)	Diffuse low-grade gliomas MAPK pathway-altered	BRAF, FGFR1, FGFR2, NTRK fusions, MET alterations	MAPK/ERK	**Weak to moderate (context-dependent)**-BRAFV600E and KIAA1549-BRAF fusion driven MAPK activation can affect LATS 1/2 phosphorylation status-NTRK1 has been shown to facilitate tumor cell proliferation by positively regulating YAP -MET-mediated cytoskeletal remodeling and actin-dependent mechanotransduction, may influence Hippo activity	RAS-driven MAPK signaling activates the PI3K-AKT-mTOR pathway, which may cooperate with YAP/TAZ signaling	[110–113, 117]
Pediatric-type diffuse high-grade gliomas (pDHGGs)	Diffuse midline glioma, H3k27-altered Diffuse midline glioma, H3G34-altered Diffuse midline glioma, H3-wt and IDH-wt	H3K27M, H3G34R/V PDGFRA, EGFR and TERT alterations, MYCN amplification	Epigenetic dysregulation + RTK	**Moderate (functional evidence)** -YAP–TEAD-mediated recruitment of the NuRD complex promotes histone deacetylation, including loss of H3K27ac at regulatory regions, highlighting a role for Hippo in chromatin remodeling—YAP knockdown can reduce EGFR signaling-YAP contributes to resistance to EGFR inhibitors -EGFR signaling can directly activate YAP-YAP knockdown can cause downregulation of MYC target genes	SWI/SNF chromatin remodeling counteracts Polycomb-mediated chromatin repression and modifies YAP/TAZ-mediated transcription	[114–122]
Ependymomas	Supratentorial	YAP fusion (commonly YAP1- MAMLD1 fusion)	Hippo	**Strong (direct genetic activation)**-YAP1 fusion results in activation of YAP signaling		[117, 123]
		RELA-ZFTA fusion,	NF-κB	**Moderate (functional evidence)** -MST1 can downregulate NF-κB signaling	JAK-STAT pathway involvement as NF-κB-driven cytokines activate STAT-3, which also interacts with YAP/TAZ to promote tumorigenic effects	[117, 124, 125]

### Validation of Hippo-pathway associated vulnerabilities in pediatric brain cancers

In addition to transcriptomic analyses, functional genomic datasets provide an important orthogonal strategy to interrogate the role of Hippo signaling in pediatric brain tumors. The Pediatric Cancer Dependency Map represents a particularly valuable resource, integrating CRISPR-Cas9 loss-of-function screens across a diverse panel of pediatric cancer models, including multiple brain tumor subtypes. We have performed a comprehensive query of YAP1 in the DepMap portal (DepMap Public 26Q1, Chronos scorin [126], which incorporates the first-generation Pediatric Cancer Dependency Map (PedDep v.1) generated across 13 pediatric solid and brain tumor types, alongside broader adult cancer data from over 1200 cell lines.

Regarding the overall YAP1 dependency profile, YAP1 was classified as strongly selective, with dependency (gene effect score <  − 0.5) observed in 370 out of 1208 screened cell lines by CRISPR and in 47 out of 712 lines by RNAi. The CRISPR predictability score reached the 99.1th percentile -the highest tier for mechanistically interpretable dependencies- indicating that YAP1 dependency is highly predictable from molecular features. The top genetic co-dependencies of YAP1 included TEAD1 (Pearson r = 0.27) and TEAD3 (r = 0.27), directly confirming that the YAP–TEAD transcriptional complex is the functionally relevant node, as we describe throughout the manuscript. The strongest negative co-dependencies -NF2 (Merlin; r = −0.24 CRISPR, −0.32 RNAi) and LATS2 (r = −0.23 CRISPR)- confirm that inactivation of upstream Hippo tumor suppressors renders cells selectively dependent on YAP1 for survival, as described in Sects. 3. The top predictive biomarker for YAP1 dependency across the dataset was the expression of WWTR1 (TAZ) (relative importance 18%), consistent with co-activation of the YAP/TAZ axis as the molecular context that defines this vulnerability.

The perturbation effect analysis across the 91 available CNS/Brain cell lines revealed a context-dependent pattern of YAP1 dependency, consistent with the well-documented molecular and biological heterogeneity of these tumors. While the median gene effect across adult-type diffuse glioma lines was approximately −0.25 to −0.35 (indicating moderate group-level dependency), a clinically relevant subset of glioma and pediatric embryonal tumor cell lines exhibited strong YAP1 dependency (gene effect score ≤ −0.5): among adult-type diffuse glioma lines, these included AM38 (−1.44), M059K (−0.82), U178 (−0.73), SW1088 (−0.70), HS683 (−0.68), SNU1105 (−0.65), DKMG (−0.64), YH13 (−0.61), and KNS81 (−0.61); among pediatric embryonal tumor lines, strong dependency was observed in BT12 (−1.36), CHLA266 (−1.16), and BT16 (−0.79). The degree of YAP1 dependency is therefore context-dependent and consistent with the oncogenic background of each line (Supplementary Fig. 1). The most strongly YAP1-dependent glioma cell lines (e.g., AM38, M059K) are known to harbour constitutive activation of MAPK or PI3K signalling, in agreement with the findings of Mayrhofer et al. [91] that MAPK/PI3K-driven tumours require persistent nuclear YAP for high-grade progression.

### Preclinical models of pediatric brain tumors: implications for Hippo-targeted therapies

Preclinical models (cell lines, patient-derived cell cultures and xenografts, tumor-brain organoids and genetically engineered models) are indispensable tools to comprehend the mechanisms of the development and progression of the disease [127]. Currently, several *in vivo* models are available for disease monitoring as well as the development of efficient and targeted Hippo signaling therapeutic approaches. He et al. has generated 21 patient-derived pHGG orthotopic xenograft (PDOX) models by implanting human cancer cells into the brain of immunodeficient mice. In a subset of these tumors, dissociated cells were cultured to produce eight matched cell line models, classified according to DNA methylome profiling. It was shown that the PDOXs models and matched cell lines were able to recapitulate recurrent mutations and characteristic expression signatures of pHGG. They also represented rare tumor subtypes with limited available models, including H3.3 G34R glioblastomas, pHGG with MMRD, tumors of the pedRTKIII subgroup and PXAs. However, there were also molecular discrepancies between the tumors of origin and the matched preclinical model, probably reflecting the tumor heterogeneity between the samples analyzed and the cells that were implanted [127].

In addition, researchers from the St. Jude Children’s Research Hospital have developed 37 PDOX models generated from the treated patients with frequent and also rare tumor entities [128]. Such patient-derived xenograft models recapitulate tumor architecture and microenvironmental stimuli to assess Hippo-driven tumor features. The generated cells also retain several molecular alterations from the initial tumors, which converge on YAP/TAZ signaling, thereby promoting therapeutic resistance. Furthermore, several patient-derived organoids have been developed from ependymomas, medulloblastomas and low-grade tumors with the capacity to produce xenografts. These preclinical models showed responses consistent with those observed in patients under the same treatments [129].

Tumor-specific preclinical models have also been developed. In the study of Grasso et al., patient-derived DIPG cultures were established for drug sensitivity screening, as well as orthotopic xenografts to test effective treatment strategies [130]. Finally, a relevant preclinical zebrafish model to Hippo signaling which expresses oncogenes that trigger MAPK and PI3K signaling in neural progenitor cells is also particularly valuable. As previously described, this model exhibits enhanced YAP activation differentiating mesenchymal glioblastomas from low-grade tumors [91]. Taken altogether these preclinical models present valuable tools for future investigation of Hippo-associated molecular mechanisms and novel treatment strategies, being able to recapitulate the tumors’ heterogeneity and microenvironment status.

## Therapeutic targeting strategies for the Hippo pathway

Several strategies have been exploited to target the activation of the Hippo signaling pathway in disease context, including inhibitors of YAP/TAZ-TEAD transcriptional activity, activators of upstream Hippo kinases (MST/LATS), modulators of mechanical and cytoskeletal inputs as well as G-protein coupled receptor (GPCR) and inhibitors of other YAP/TAZ co-factors (Table 2).

**Table 2:** Hippo pathway targeting strategies

Targeting Strategy	Compounds	Effects	Reference
**Inhibition of YAP/TAZ–TEAD transcriptional activity**			
Combined inhibition of TEAD palmitoylation	VT104/107, MGH-CP1, JM7	- suppresses *NF2*-deficient mesothelioma - suppress stemness, cell proliferation, colony formation and migration in breast, ovarian and mesothelioma cell lines	[132–137]
Direct disruption of YAP/TAZ–TEAD protein–protein interaction	Peptide 17, vertpeforfin	- reduced tumor size and cell proliferation in mice inoculated with tumor cells - suppress cell growth and proliferation in human glioma cells	[138–146]
**Reactivation of upstream Hippo kinases (MST/LATS)/Suppression of negative regulators of the pathway**	ormin/statins	- induce oxidative stress, activate AMPK and lead to activation of LATS1/2	[147, 148]
**Modulation of mechanical and cytoskeletal inputs**	ROCK inhibitors	- reduce actomyosin contractility and cytoskeletal tension, inhibiting YAP/TAZ nuclear translocation	[29]
**GPCR modulation**	Agonists of Gs-coupled GPCRs (epinephrin, dobutamine, dihydrexidine)	- epinephrine induces YAP/TAZ phosphorylation in breast cancer cells and in mouse heart - dobutamine phosphorylates YAP/TAZ in an osteoblastoma cell line - dihydrexidine (DHX) activates the dopamine D1 receptor, increasing intracellular cAMP levels which activate the GTPase RAP2, reducing YAP/TAZ activity	[44, 149, 150]
**Targeting YAP/TAZ co-factors beyond TEAD**	JQ1	- suppresses YAP expression *in vitro* and YAP-dependent carcinogenesis *in vivo*	[151]
**Indirect inhibitors**	XAV939, ERK1/2 inhibitors, PI3K inhibitors	- XAV939 blocks YAP activity by preserving the stability of AMOT proteins - ERK1/2 inhibitors suppress YAP protein expression and induce degradation of YAP in human lung carcinoma cells - PI3K inhibitors increase LATS1 and Mob1 and reduce YAP protein levels in human colorectal carcinoma cells	[51, 152, 153]

### Inhibition of YAP/TAZ–TEAD transcriptional activity

Even though YAP/TAZ play an important role in oncogenesis, they are hard to target directly due to their intrinsically disordered nature. Binding of YAP1/TAZ to TEAD transcription factors has been shown to serve as a critical point in the Hippo pathway–dependent transcription and due to its ease of targeting, it has become the most well-developed therapeutic axis [131].

#### TEAD palmitoylation inhibitors

TEAD proteins undergo enzyme-independent palmitoylation (attachment of palmitate) at a conserved cysteine residue under physiological conditions [132]. This palmitoylation helps stabilize TEAD and is important for its transcriptional activity. It also promotes membrane attachment and co-factor binding of proteins [133]. Consequently, small molecules have been developed that bind this pocket and block TEAD palmitoylation, leading to reduced stability or altered conformation of TEAD, thereby impairing its interaction with YAP/TAZ [131]. Tang et al. introduced VT104/107, which were covalent pan-TEAD auto-palmitoylation inhibitors and were the first to demonstrate that blocking TEAD palmitoylation suppresses NF2-deficient mesothelioma, a YAP/TAZ-driven tumor [134]. Sun et al. tested the effects of MGH-CP1 and its analogues, reversible TEAD palmitoylation inhibitors, *in vivo* and *in vitro* and demonstrated that while they suppress stemness, cell proliferation and colony formation, they do not induce cell death. Furthermore, blockage of TEAD leads to VGLL3-driven transcriptional activity of the SRY-box transcription factor 4 (SOX4)/PI3K/AKT signaling axis, leading to therapeutic resistance to the aforementioned inhibitors. Accordingly, the use of TEAD inhibitors and AKT inhibitors can have a synergistic anticancer effect [135]. Gridnev et al. identified JM7, another TEAD palmitoylation inhibitor and destabilizing agent, which has been shown to inhibit the expression of YAP target genes and suppress cell growth, colony formation, and migration without altering YAP/TEAD localization in breast, ovarian and mesothelioma cell lines [136].

#### Direct disruption of YAP/TAZ–TEAD protein–protein interaction (PPI)

Interaction of the TEAD–YAP complex is facilitated through three distinct structural interfaces. While interface 1, facilitated by seven hydrogen bonds, plays a marginal role in the binding, interfaces 2, a hydrophobic pocket, and especially 3, a hydrophobic region coil region of YAP interacting with TEAD, contribute greatly to the binding; thus, peptides and small molecule inhibitors targeting these interfaces have been developed [137–140]. Zhang et al. identified Peptide 17, a peptide which blocks the YAP/TAZ–TEAD binding. When injected in mice inoculated with tumor cells, peptide 17 reduced tumor size and cell proliferation [65, 141].

Incidentally, verteporfin, a benzoporphyrin derivative used as a drug to treat macular degeneration is in its on-photoactivated form a YAP/TAZ–TEAD PPI inhibitor which can suppress cell proliferation of human glioma cells [142–144].

### Activation of upstream Hippo kinases (MST/LATS)

Reactivating the canonical Hippo kinase cascade provides an alternative mechanism, by which YAP/TAZ can be phosphorylated and inactivated. The most direct approach involves kinase activation with small molecules that enhance MST1/2 or LATS1/2 phosphorylation; however, such activators have proven hard to develop. Incidentally, formin has been shown to induce oxidative stress, activating the AMP-activated protein kinase (AMPK) pathway, which eventually leads to activation of LATS1/2 [145].

A different approach involves the suppression of negative regulators of the pathway. For example, statins indirectly activate LATS1/2 by reducing geranylgeranyl pyrophosphate (GGPP) levels, a LATS1/2 inhibitor [146].

### Modulation of mechanical and cytoskeletal inputs

Dupont et al. demonstrated that alterations in ECM density and cytoskeletal tension can affect YAP/TAZ activity. Therefore, it was suggested that drugs which modify these upstream inputs could have therapeutic value. A representative example is ROCK inhibitors, which reduce actomyosin contractility and cytoskeletal tension, consequently inhibiting YAP/TAZ nuclear translocation [29].

### GPCR modulation

In preclinical models, agonists of GPCRs, such as epinephrine, can trigger YAP/TAZ phosphorylation in breast cancer cells as well as in the mouse heart, a primary target organ of epinephrine [44]. Similar YAP/TAZ phosphorylation has been observed with dobutamine, a β1-adrenergic agonist, in an osteoblastoma cell line, occurring independently of LATS1/2 phosphorylation [147]. Likewise, dihydrexidine (DHX), a dopamine D1 receptor agonist, activates the dopamine D1 receptor, raising intracellular cAMP levels. This stimulates the cAMP sensors EPAC1 and EPAC2, which activate the GTPase RAP2. RAP2 then triggers MAP4K7, which reduces YAP/TAZ activity independently of MST1/2 [148].

### Targeting YAP/TAZ co-factors beyond TEAD

Song et al. showed that there is a positive correlation between bromodomain-containing protein 4 (BRD4), a chromatin reader member of the BET family, and YAP/TAZ expression. Pharmacological inhibition of BRD4 with JQ1 suppresses both YAP expression *in vitro* and YAP-dependent carcinogenesis *in vivo* [149].

### Indirect inhibitors

Tankyrases, which belong to the Poly(ADP-ribose) polymerase (PARP) family, bind to angiomotin family proteins (AMOT) and induce their ligase-dependent degradation. Wang et al. showed that AMOT proteins suppress YAP-mediated transcription by promoting YAP cytoplasmic localization and that the use of XAV939, a tankyrase inhibitor, blocks YAP activity by preserving the stability of AMOT proteins [150].

Extracellular signal-regulated kinases 1/2 (ERK1/2) are core constituents of the MAPK pathway. The administration of ERK1/2 inhibitors has been shown to suppress YAP protein expression and induce degradation of YAP in human lung carcinoma cells [51].

In human colorectal carcinoma cells, PI3K inhibitors have been shown to increase LATS1 and MOB1 and reduce YAP protein levels [151].

### Hippo targeting agents in clinical trials

Most Hippo pathway–targeted therapies remain in preclinical development, and only a limited number of drugs targeting the YAP/TAZ–TEAD transcriptional axis or YAP directly have entered clinical trials. These include VT3989, a TEAD palmitoylation inhibitor in phase 1/2 trials being studied on refractory solid tumors, with an emphasis on mesothelioma. Reported side effects including reversible decreases in renal function [152]. Another such example is IK-930, an oral selective TEAD inhibitor previously used in Phase I dose-escalation studies for NF-2 deficient solid tumors, as well as hemangioendotheliomas with TAZ-CAMTA1 or YAP1-TFE3 gene fusions, that was, however, terminated due to sponsor specific reasons [153–155]. IAG933 is a disruptor of the YAP/TAZ–TEAD PPI in phase 1 trial being tested on patients with mesothelioma as well as other solid tumors with NF2/LATS1/LATS2 mutations or functional YAP/TAZ fusions [156]. In addition to these TEAD-targeting drugs, ION537, an antisense oligonucleotide targeting YAP1, was tested on patients with advanced solid tumors in a completed phase 1 trial [157].

Overall, clinical application of Hippo pathway inhibition is still in its early but promising phase, with TEAD-targeting drugs attracting most of the attention.

## Limitations

The limited number of studies directly investigating the functional role of the Hippo signaling pathway in pediatric gliomas makes it difficult to draw definitive conclusions. Furthermore, the wide heterogeneity of pediatric glioma subtypes, together with the historical differences in tumor classification among older studies, complicates the interpretation and comparison of findings, particularly those related to YAP/TAZ signaling. Although this review incorporates substantial evidence from single-cell transcriptomic and genomic studies, it is a narrative review rather than a formal meta-analysis and thus, a selection bias cannot be fully excluded. Finally, the rarity of several pediatric glioma subtypes reduces the reproducibility and statistical power of individual studies, limiting the ability to derive broadly generalizable conclusions.

## Conclusion and future perspectives

Emerging evidence suggests that the Hippo signaling pathway plays an active role in the development and progression of pediatric gliomas. Although the pathway has not yet been comprehensively investigated in this context, dysregulation of key Hippo effectors, particularly YAP and TAZ, have been shown to contribute to tumor cell proliferation, resistance to apoptosis, and increased tumor aggressiveness. These findings highlight the significance of the Hippo pathway as a promising research field for identification of diagnostic biomarkers and potential therapeutic targets in pediatric gliomas [158]. While several studies have explored Hippo pathway modulation as a therapeutic strategy in adult gliomas [159], substantial gaps still remain in translating these findings to pediatric tumors.

Future studies should focus on integrating the growing body of transcriptomic and genomic data available for pediatric brain tumors in order to better define the molecular interactions and functional relevance of Hippo signaling within the unique pediatric glioma microenvironment [160]. In this regard, the 22-gene YAP/TAZ transcriptional target score developed by Wang et al. (2018), which includes validated downstream targets such as *CTGF*, *CYR61*, *AXL*, *ANKRD1*, *AMOTL2* and *NUAK2*, represents a valuable computational tool for assessing YAP/TAZ transcriptional activity across tumor types [161]. Applying this signature to pediatric brain tumor datasets, including those from the Open Pediatric Brain Tumor Atlas (OpenPBTA), may facilitate quantitative stratification of low-grade and high-grade gliomas and help identify molecular subgroups characterized by increased Hippo pathway dysregulation.

In parallel, emerging single-cell transcriptomic resources offer important opportunities for future investigation. The Pediatric Neuro-Oncology Cell Atlas provides single-cell data from both pediatric low-grade and high-grade gliomas and may enable comparative analyses of Hippo pathway activity across glioma subtypes and cellular populations. In addition, the Childhood Cancer Data Lab’s Single-cell Pediatric Cancer Atlas (ScPCA), which contains curated single-cell and single-nucleus RNA sequencing datasets from multiple pediatric malignancies, represents an additional resource for studying Hippo pathway effector expression at single-cell resolution. Integration of these large-scale datasets into future studies will be essential for improving our understanding of Hippo signaling in pediatric glioma biology and for identifying clinically relevant therapeutic vulnerabilities.

## Supplementary Information

Below is the link to the electronic supplementary material.

ESM1 (PDF 60.6 KB)

## Data Availability

Not applicable.
