# Supplementary material for: The Hippo signaling pathway in pediatric brain tumors: molecular mechanisms and therapeutic opportunities
Source: Cancer Metastasis Rev. 2026 Jun 13;45(2):40. doi: 10.1007/s10555-026-10349-8 (PMC13264582; doi:10.1007/s10555-026-10349-8)
Supplement: Supplementary file 1 — (PDF 60.6 KB) [file 10555_2026_10349_MOESM1_ESM.pdf]

Supplementary Figure S1
YAP1 CRISPR dependency across CNS/Brain cancer cell lines (DepMap Public 26Q1)

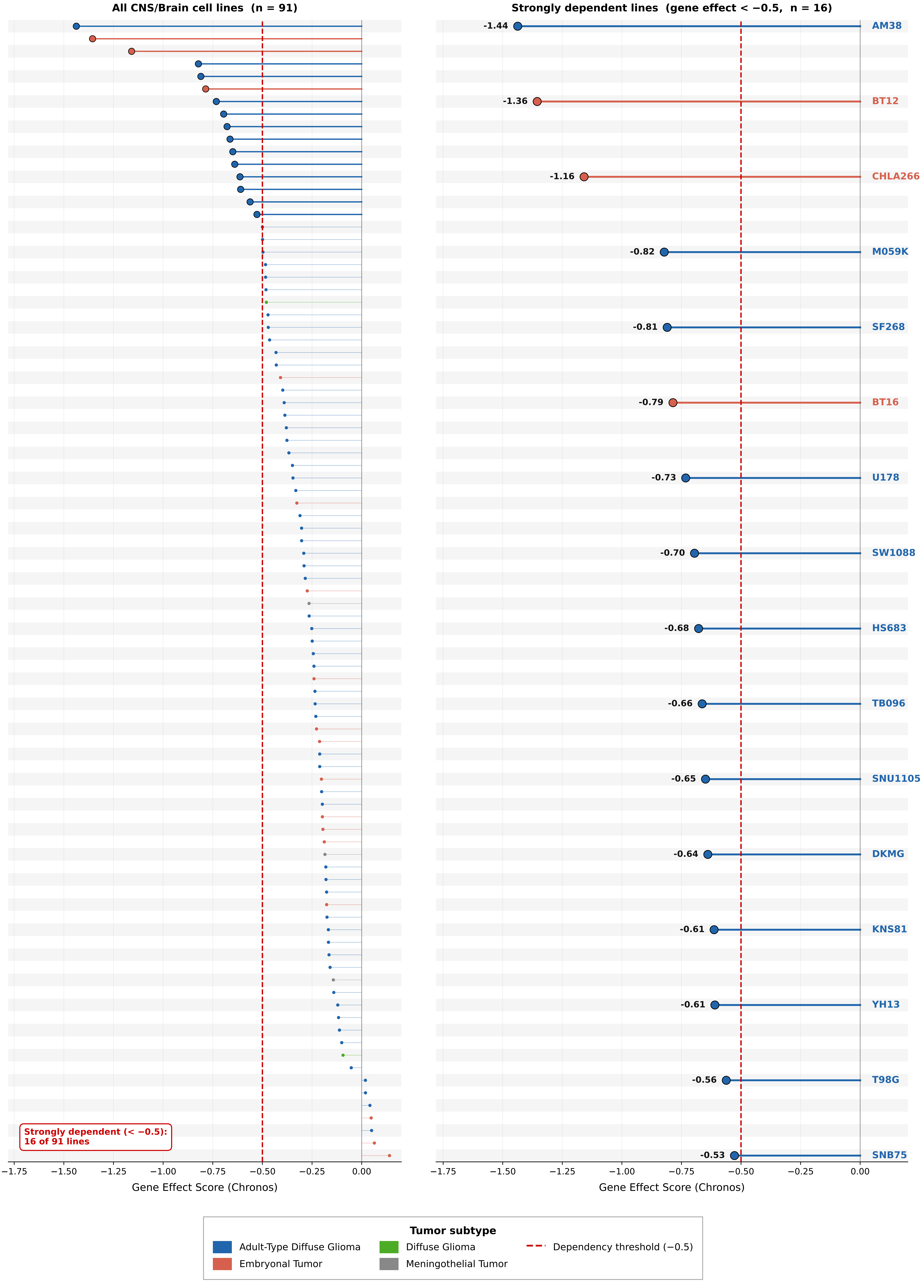

Source: DepMap Public 26Q1 (Chronos scoring). Dharia NV et al., Nat Genet 2021; DOI: 10.1038/s41588-021-00819-w
